# Signs and Symptoms of Temporomandibular Joint Disorders Related to the Degree of Mouth Opening and Hearing Loss

**DOI:** 10.1186/1472-6815-11-5

**Published:** 2011-05-25

**Authors:** Panagiotis Kitsoulis, Aikaterini Marini, Kalliopi Iliou, Vasiliki Galani, Aristides Zimpis, Panagiotis Kanavaros, Georgios Paraskevas

**Affiliations:** 1 Orthopaedic Surgeon, Professor of Anatomy-Histology-Embryology, Medical School, University of Ioannina, Greece; 2 Intern of Internal Medicine, Assistant in the Laboratory of Anatomy-Histology-Embryology, Medical School, University of Ioannina, Greece; 3 Assistant in the Laboratory of Anatomy-Histology-Embryology, Medical School, University of Ioannina, Greece; 4 Associate professor of Anatomy-Histology-Embryology, Medical School, University of Ioannina, Greece; 5 Orthopaedic Surgeon, Lecturer of Anatomy, Medical School, University of Larissa, Thessaly, Greece; 6 Professor and Chairman of Anatomy-Histology-Embryology, Medical School, University of Ioannina, Greece; 7 Orthopaedic Surgeon, Assistant Professor of Anatomy, Medical School, Aristotle University, Thessaloniki, Greece

**Keywords:** temporomandibular disorders, mouth opening, aural symptoms, hearing loss, audiometry

## Abstract

**Background:**

The temporomandibular joint is a unique bi-condylar joint involved in mastication and speech. Temporomandibular joint disorders (TMD) have a range of symptoms, including aural symptoms, and are present in approximately 75% of normal populations. The present study examined the relationship between signs and symptoms of TMD and mouth opening, gender, joint and aural symptoms, and hearing loss.

**Methods:**

The study involved 464 healthy Greek university students (156 men and 308 women) with a mean age of 19.6 years. Age, gender and maximum mouth opening was recorded. Mouth opening was measured using Vernier calipers. An anamnestic questionnaire was used to stratify the subjects into four groups based on TMD severity. Aural symptoms and an audiogram were recorded for each subject too. Data were analyzed using multifactor ANOVA, chi-square, t-test, Mann-Whitney and Kruskal-Wallis tests.

**Results:**

The overall incidence of TMD signs and symptoms was 73.3%. The incidence and severity was greater in females than males (p-value 0.0001 < 0.05). The number of aural symptoms was associated to the TMD severity (p-value 0.0001 < 0.05) as well as maximum mouth opening (p-value 0.004 < 0.05). Audiometry showed that moderate and severe TMD was associated with hearing loss of median and low tones respectively (p-value 0.0001 < 0.05). TMJ pain (p-value 0.0001 < 0.05), TMJ ankylosis (p-value 0.0001 < 0.05), bruxism (p-value 0.0001 < 0.05) and ear itching (p-value 0.0001 < 0.05) were also found to be statistically different between TMD and non-TMD subjects.

**Conclusions:**

TMD signs and symptoms were more common and severe in females than males. TMD severity is correlated with the degree of mouth opening and the number of aural symptoms. The absence or presence of mild TMD are associated with normal audiograms while moderate and severe TMD are related to hearing loss in median and low tones respectively. Bruxism, joint ankylosis, joint pain and ear itching were more common in TMD than non-TMD patients.

## Background

The temporomandibular joint is critical for normal mouth function, and thus plays a role in chewing, swallowing, speaking, oral health and nutrition. The temporomandibular joint is a synovial joint containing an articular disk which allows for hinge and sliding movements. The articular surfaces are covered by avascular and non-innervated fibrocartilage which has a high regenerative capacity. The temporalis and masseter muscles control joint's motion.

The term temporomandibular disorders (TMD) is a collective one, representing a sub-classification of muscle-skeletal disorders, and more specifically a number of signs and symptoms involving the masticatory muscles, the temporomandibular joint and associated structures. It is estimated that about one third of adults have TMD symptoms [1]. TMD has been related to stress, age, gender, personality and other systematic factors [2].

The maximum mouth opening distance is a generally accepted measurement to estimate temporomandibular joint mobility and function [3]. Mouth opening can be measured using gauges or calipers, and while the normal range differs between populations, the critical functional opening is 35-40 mm [4,5].

Aural symptoms such as tinnitus, otalgia, dizziness or vertigo, otic fullness sensation, hyperacousia or hypoacousia are thought to be associated with TMD [6,7], while their incidence reaches 85% in TMD patients [8]. A causative role of TMD in otic symptomatology remains a matter of debate. Several studies have shown that aural symptoms may have no otic origin [9]. Theories on the etiology of aural symptoms are based mainly on the common embryologic origin of the temporomandibular joint and the middle ear from Meckel cartilage, the presence of structures that connect the middle ear with the temporomandibular joint and the common innervation of the masticatory muscles and the ear.

The present study examined 464 healthy young Greek adults for the presence of TMD signs and symptoms. The study analyzed data for probable correlations between TMD severity and range of mouth opening, aural and joint symptoms and hearing loss. A feature of the study was the use of audiography to objectively assess the co-existence of hearing loss and TMD.

## Methods

### Material

The study was performed in the Laboratory of Anatomy at the Medical School of the University of Ioannina. The project involved 464 volunteers (156 males and 308 females) aged from 18 to 26 years (mean age 19.6 years) who attending the University of Ioannina. All volunteers were healthy, without active disease or craniofacial anomalies.

Maximum mouth opening (mm) was measured using a 1 mm calibrated Vernier caliper. Subjects opened their mouths as wide as possible, and the inside jaws of the caliper were used to measure the interincisal distance (the distance between the edges of the upper and lower central incisors). During measurements, subjects were seated in a chair with head supported and held in the anatomical position in order to control for additional movements of the cervical, thoracic and lumbar spine [10]. Only the maximum active mouth opening was measured in order to avoid errors due to different applied force during passive opening [11]. Each measurement was repeated and assessed twice under the supervision of two observers.

At the completion of the measurement, the gender and age of each subject was recorded. Temporomandibular symptoms were assessed using a validated self-report anamnestic questionnaire composed of 10 questions [12]. The questionnaire asked about any history of difficulties in opening the mouth or moving the mandible sideways, discomfort while chewing, headaches, neck and shoulder pain, otalgia, temporomandibular joint sounds, abnormal biting, unilateral chewing and facial pain. We relied on the subject's sense of joint sounds, as referred elsewhere [13]. The possible questionnaire answers were ''YES'', ''NO'' and ''SOMETIMES'', and subjects were directed to choose only one of those answers for each question. Scores were obtained based on those answers. For each ''YES'' the score was ''2'', while for each ''NO'' or ''SOMETIMES'' the score was ''0'' and ''1'' respectively. For bilateral ear pain or temporomandibular joint sounds one more point was added and one more for intense headaches. Those scores were used to create four TMD groups: non- (score 0-3), mild (score 4-8), moderate (score 9-14) or severe (score 15-23) [12]. In addition to the questionnaire, subjects were asked about three further probable TMD related symptoms: bruxism, temporomandibular joint ankylosis and history of joint pain. Joint ankylosis was evaluated only if addressed to a physician for that reason.

Using another questionnaire, subjects were asked whether they had experienced the following aural symptoms: a feeling of fullness or pressure in ear, brief vertigo (less than 5 minutes), cold and warm air sensitivity, itching of the acoustic canal, and a history of diagnosed hearing loss, more than four events of otitis, tinnitus or ear pain. Subjects were categorized based on having none or less than two of those symptoms, more than two of the above symptoms, and more than four of the aforementioned symptoms.

After completing questionnaires, the volunteers were taken to a separate room where tympanometry and pure tone audiography were conducted to objectively evaluate hearing loss. Pure tone audiography included tones between 125-8000 Hz. All subjects were found to be normal in terms of tympanometry results [14]. Each audiography pattern was then interpreted according to the Rösel calculator as a percentage of hearing loss for each ear [15]. Subjects were categorized according to five types of quantitative hearing loss: ≤15% in both ears, > 15% but < 20% in one ear, > 15% but < 20% in both ears, ≥20% in one ear and ≥20% in both ears. They were also categorized into five groups based on qualitative hearing loss according to audiological findings: no hearing loss, loss of low tones (125-250 Hz) in one and in both ears, unilateral loss of median tones (25-4000 Hz) and bilateral loss of median tones. No participant had a loss of high tones (4000-8000 Hz) hearing, hence no such category was required.

All subjects gave written informed consent regarding participation and publication of the data. The study was approved by the Ethics Committee of the Medical School of University of Ioannina.

### Statistical analysis

Our variables for analysis were the age, the gender, the number of aural symptoms, range of mouth opening, and the degree of qualitative and quantitative hearing loss. ANOVA and t-tests were used to compare normally distributed variables, and Kruskal-Wallis and Mann-Whitney tests were used for comparing non-normally distributed variables (i.e. mouth opening in TMD and non-TMD subjects, mouth opening and gender, mouth opening and TMD severity). Chi-square test was used to compare both normally and non-normally distributed variables. We also investigated for correlations between TMD severity and bruxism, joint ankylosis, joint pain and ear itching using chi-square testing.

Analysis was performed on anonymous data using SPSS version 18. Kolmogorov-Smirnov and Shapiro-Wilk tests of normality were employed to determine data distribution. The level of statistical significance was p < 0.05. The confidence interval was 95%.

## Results

Of the 464 study subjects, 73.3% were diagnosed with TMD, and 26.7% were diagnosed as not having TMD. Of the 340 TMD subjects, TMD was classified as mild in 31.76%, moderate in 40% and severe in 28.2%. In addition, the incidence and severity of TMD was greater in women than men (p < 0.05). The mean mouth opening was 46.56 mm in men and 44.43 mm in women (p < 0.05; Mann-Whitney U test). The range of mouth opening was greater in men than women (Tables 1, 2 and 3).

**Table 1:** Presence of temporomandibular joint disorders and mouth opening distance in men and women.

			TMD group				Mouth opening (mm)		
			
			non	mild	moderate	severe	N	Mean	Std. Deviation
Gender	male	Count	56	48	32	20	156	46.5641	7.21931
	
	female	Count	68	60	104	76	308	44.3377	5.82617

**Table 2:** Chi-square test reveals statistically significant relation of sex and TMD incidence and severity.

Chi-Square Tests			
	**Value**	**df**	**Asymp. Sig. (2-sided)**

Pearson Chi-Square	26.309^a^	3	.000

Likelihood Ratio	26.739	3	.000

Linear-by-Linear Association	22.306	1	.000

N of Valid Cases	464		

a. 0 cells (,0%) have expected count less than 5. The minimum expected count is 32,28.

**Table 3:** Mann-Whitney reveals a statistically significant difference of mouth opening between men and women.

**Test Statistics**^**a**^	
	**Mouth opening**

Mann-Whitney U	19240.000

Wilcoxon W	66826.000

Z	-3.512

Asymp. Sig. (2-tailed)	.000

a. Grouping Variable: gender

The mean mouth opening for the overall population was 45.08 mm. The mean mouth opening was 46.45 mm in the non-TMD, and 44.59 mm in the TMD group (p < 0.05; Mann-Whitney U test). Analysis of mouth opening distance in the four TMD groups showed that the mean mouth opening was 46.45 mm in the non-TMD group, in the mild TMD group 45.37 mm, 44.07 and 44.43 mm in the moderate and severe TMD groups respectively. Kruskal -Wallis analysis indicated that mean mouth openings differed significantly between the TMD groups (p < 0.05) (Tables 4, 5 and 6).

**Table 4:** Mouth opening distance in non-TMD, TMD subjects and TMD subgroups. Descriptive statistics.

	TMD	N	Mean	Std. Deviation
Mouth opening (mm)	Non-TMD	124	46.4476	7.16257

	TMD	340	44.5891	6.11675

	Mild	108	45.3704	5.85908

	Moderate	136	44.0792	5.69746

	Severe	96	44.4346	6.93428

**Table 5:** Mann- Whitney test shows a statistically significant difference of mouth opening between non-TMD and TMD subjects.

**Test Statistics**^**a**^	
	**Mouth opening**

Mann-Whitney U	5361.000

Wilcoxon W	9894.000

Z	-2.874

Asymp. Sig. (2-tailed)	0.005

a. Grouping Variable: TMD vs non-TMD

**Table 6:** Kruskal-Wallis reveals a statistically significant difference of mouth opening among TMD groups.

**Test Statistics**^**a,b**^	
	**Mouth opening**

Chi-square	12.368

D	3

Asymp. Sig.	.004

a. Kruskal Wallis Test

b. Grouping Variable: TMD

Pain in the temporomandibular joint was reported by 96 subjects. It was experienced by 3% of the non-TMD group, 11.1% of the mild group, 8.8% of the moderate group and 70.8% of the severe TMD group. Chi-square test showed that temporomandibular joint pain was more likely to be present in the TMD than non-TMD patients (p < 0.05) History of temporomandibular joint ankylosis was reported by 52 subjects. It was referred by 3.7% of mild, 2.9% of moderate and 45.8% of severe TMD subjects. No non-TMD participants had joint ankylosis. Chi-square test found that joint ankylosis was more likely to be found in TMD patient than non-TMD subjects (p < 0.05). Bruxism was reported by 80 subjects. It was recorded in 0% of non-, 7.4% of mild, 29.4% of moderate, and 33.3% of severe TMD patients. Chi-square test showed that bruxism was more likely to occur in TMD than non-TMD subjects (p < 0.05). Ear itching was reported by 92 subjects. It was experienced by 12.9% of non-, 11.1% of mild, 26.4% of moderate and 29.1% of severe TMD subjects. Chi-square test revealed that ear itching differed between TMD groups (p < 0.05). Ear itching was commoner in TMD than non-TMD subjects (Tables 7, 8).

**Table 7:** Presence of temporomandibular joint ankylosis, bruxism, aural symptoms, pain and ear itching according to TMD group.

TMJ pain *,ankylosis,bruxism,aural symptoms,pain TMD Crosstabulation							
			TMD group				
			
			non	mild	moderate	severe	Total

Ankylosis	No	Count	124	104	132	52	412
	
	Yes	Count	0	4	4	44	52

Bruxism	No	Count	124	100	96	64	384
	
	Yes	Count	0	8	40	32	80

Aural Symptoms	0-2 aural symptoms	Count	92	76	76	44	288
	
	> 2 aural symptoms	Count	28	28	52	24	132
	
	> 4 aural symptoms	Count	4	4	8	28	44

Pain	No	Count	120	96	124	28	368
	
	Yes	Count	4	12	12	68	96

Ear itching	No	Count	108	96	100	68	372
	
	Yes	Count	16	12	36	28	92

**Table 8:** Chi-square tests show a statistically significant relation of TMJ ankylosis, bruxism, number of aural symptoms, pain, ear itching and TMD severity.

Chi-Square Tests	
**TMJ symptom/sign**	**p-value**

Ankylosis	0.000

Bruxism	0.000

Aural Symptoms	0.000

Pain	0.000

Ear Itching	0.000

Subjects were assessed for their aural symptoms. We found that 62% had no or less than two symptoms, 28.44% had > 2 symptoms, and 9.4% had > 4 symptoms. We analyzed the number of aural symptoms in relation to TMD severity. The majority of the non-TMD (74.2%) or mild TMD (70.4%) subjects had no aural symptoms. For the moderate TMD group, 55.9% had none and 38.2% had more than two aural symptoms. In the severe TMD group, 25% had more than two and 29.2% had more than four aural symptoms. Chi-square test showed that the severity of TMD is related to the number of aural symptoms. Qualitative assessment showed that 51.7% of the subjects had normal hearing, 13.8% and 18.1% of the subjects had unilateral and bilateral low tones loss respectively, and 8.6% and 7.8% of the subjects had bilateral and unilateral median tones loss respectively. We analyzed qualitative hearing loss and TMD groups using chi-square test. We found that TMD severity was correlated with hearing loss (p < 0.05). Most subjects without hearing loss belonged to the non-TMD group (46.6%), while most subjects with low tones hearing loss in both ears belonged to the severe TMD group (47.6%). For median tones, hearing loss in both ears was more common in moderate (50%) compared to severe TMD (40%) subjects. Furthermore, most mild-TMD subjects had no hearing loss (59.3%), Quantitative assessment using audiograms showed that 61.2% of the subjects had ≤15% hearing loss in both ears, 22.4% had > 15% loss in one ear, 5.2% had > 15% loss in both ears, 6.9% had ≥20% loss in one ear and 4.3% had ≥20% loss in both ears. Chi-square test showed that there was a relationship between TMD severity and degree of hearing loss (p < 0.05). Most non- and mild TMD subjects had ≤15% hearing loss. For the severe TMD group, 25% had no hearing loss, 16.7% had > 15% loss in one ear and 16.7% had > 15% loss in both ears, 20.8% had ≥20% loss in one ear, and 20.8% (n = 20) had ≥20% loss in both ears (Tables 9, 10).

**Table 9:** Quantitative hearing loss and loss of tonal hearing according to TMD group.

Tonal hearing loss * TMD Crosstabulation							
			TMD Group				
			
			Non	mild	moderate	severe	Total

Hearing loss	normal≤15%	Count	104	88	68	24	284
		
	In one	Count	16	16	56	16	104
	
	ear > 15%						
		
	In one	Count	4	0	8	20	32
	
	ear≥20%						
		
	In both	Count	0	4	4	16	24
	
	ears > 15%						
		
	In both	Count	0	0	0	20	20
	
	ears≥20%						

Tonal hearing loss	No loss	Count	112	64	52	12	240
	
	Loss in low tones in both ears	Count	0	12	32	40	84
		
	Loss in low tones in one ear	Count	8	24	8	24	64
	
	Loss in median tones in both ears	Count	0	4	20	16	40
		
	Loss in median tones in one ear	Count	4	4	24	4	36

Tonal hearing loss, ear itching, hearing loss -TMD.

**Table 10:** Statistically significant relation of tones hearing loss, quantitative hearing loss and TMD via chi-square tests.

Chi-Square Tests	
	p-value

Quantitative hearing loss	0.000

Tonal hearing loss	0.000

## Discussion

The present study examined the relationship between TMD symptoms and signs and mouth opening, gender, aural symptoms, temporomandibular joint pain, temporomandibular joint ankylosis, bruxism and hearing loss.

We found that 73.3% of the young Greek adult population examined in this study had TMD symptoms and signs. This finding is consistent with previous studies involving college students [12,16]. Compared to previous studies on young populations, the present population had fewer subjects with mild TMD (31.76%) and more with moderate (40%) and severe TMD (28.2%).

We found that mouth opening differed, according to TMD severity, and the least opening was observed in the severe TMD group. Interestingly, we found that while mouth opening was less in severe TMD subjects (44.43 mm) compared to non-TMD subjects (46.45 mm), it was greater in severe TMD subjects than moderate TMD ones (44.08 mm). It has already been reported that joint hypermobility is often present in TMD patients [2]. Mouth opening cannot be used as a sole criterion of TMD presence and the range of mouth opening found in our population could be considered as functional. Nevertheless, for the needs of our study, we compared the values found for each stratification group even of considered normal.

Ethnicity is believed to affect the degree of mouth opening [12,17]. The present study is the first focused on a young Greek adult population. The overall mean mouth opening recorded was 45.09 mm. The only previous study of a Greek population took place in 1989 and involved participants aged between 18 and 70 years. The average maximum mouth opening was 52.85 mm for males and 48.34 mm for females [18]. Studies of other ethnicities found that the average mouth opening was 49.10 mm in Chinese (51.11 mm for those aged 20-30 years) [19], 49.8 mm in Americans aged 16-70 years [20], 47.1 mm in Nepalese population aged 18-68 years [21], 50.77 mm in French aged 18-84 years [22], and 55.9 mm in Swedes aged 18-25 years [23]. It ranged between 41-43 mm in Irish [24] and 35-61 mm in Croatians [25].

We found that TMD was more prevalent and severe in women than men. This observation is consistent with previous studies, as it has been reported that women present an increased risk of 1.5-2 or 2-9 times compared to men to develop TMD [17].

The current study found that pain in the temporomandibular joint was the most common symptom (27.05%) of TMD as reported elsewhere [2]. Temporomandibular joint pain is caused by the pathological contraction of the masticatory muscles which stimulates extravascular production of inflammation associated substances around the temporomandibular joint [26]. The incidence of pain in the present study is in accordance with the theory that TMD is more prevalent in early adulthood [27].

Joint sounds are regarded as the commonest sign of TMD (13.5%) [28], and are more frequent and severe in older populations. Joint sounds, such as clicks and friction, indicate temporomandibular joint derangement or joint disk displacements.

We found that bruxism was present in 27.02% of those with TMD and in none of the non-TMD subjects. Others reported that bruxism was present in 7.4%-27.2% of TMD subjects [16]. Bruxism, as clenching or grinding, may be a source or a perpetuating factor for TMD. Some investigators reported that non-TMD and TMD patients have different electromyographic patterns [29] while others failed to find a difference [30].

Head and neck ache may be of neurological, vascular, muscular, ligamental or bony origin. The temporomandibular joint has muscular and ligamentary connections to the cervical region forming a functional complex. Headache is regarded as the most common symptom (22%) of TMD patients [16], while 55% of chronic headache patients referred to a neurologist had signs or symptoms of TMD [31]. The muscles of the neck and trunk are reported to have a greater electromyographic activity in TMD subjects [32] sensitizing the sympathetic nerves of the autonomous nervous system and leading to headache via the trigeminal nerve.

We found that TMD severity is correlated with the number of aural symptoms. Costen, in 1934, firstly associated aural and craniosinusal symptoms with TMD, and named it Costen's or Otognathic Syndrome [9]. There are four main theories concerning the co-existence of aural and temporomandibular joint dysfunction symptoms. The first one suggests that disposition of the joint disk during jaw movement increases pressure in the Eustachian tube, the ear structures, the auriculotemporal and masseteric nerve and some branches of the deep posterior temporal nerve. The auriculotemporal nerve innervates the TMJ, the tympanic membrane, the anterosuperior part of the external ear and the tragus, and its stimulation gives the sense of otalgia [8]. We observed that 10.8% of TMD subjects reported ear pain, and that 75% of those TMD subjects had severe TMD. A number of series report ear pain in 35% of TMD patients [28], while 70% of temporomandibular joint pain cases are described as otalgia particularly during acute or subacute inflammation of the temporomandibular joint [33]. Additionally, we found that the rarely investigated symptom of ear itching was more common in TMD than non-TMD subjects, as reported elsewhere [28].

The second theory involves the tiny ligament called the discomalleolar or Pinto's ligament, which originates from the anterior malleolar process, passes through the petrotympanic fissure and inserts in the medial and posterosuperior part of the articular capsule and the disk [34,35]. In displacement of the temporomandibular joint disk, the discomalleolar ligament can lead to traction of the malleus [36], the ossicular chain and the tympanic membrane [34]. Furthermore, in disk displacement, the intralaminal vascular tissue moves forward and is trapped between the head of the condyle and the roof of the fossa, leading to oedema, leakage and fibrosis [37]. The petrotympanic fissure plays an important role in hosting the anterior malleolar ligament, the anterior tympanic artery and the chorda tympani nerve. Additionally, the petrotympanic fissure brings in contact the temporomandibular joint with the middle ear. Thus, beginning from the joint capsule, inflammation can spread to the origin of the levator and the tensor palatini muscles, and finally to the cul-de-sac over the vulnerable isthmus of the Eustachian tube, causing obstruction of the tube. A closed Eustachian tube may be responsible for the feeling of ear fullness, otalgia and serous otitis media [38]. This fissure's length and position may influence the development of otalgia, tinnitus, hearing loss and vertigo [35].

The third theory proposes that common innervation of the tensor veli palatini, tensor tympani, masseter, temporalis and pterygoid muscles by the motor nucleus of the trigeminal nerve is the underlying cause of the aural symptomatology in TMD patients [36]. Tensor tympani, tensor veli palatini and stapedial muscles are called auditory muscles, while the tensor veli palatini and the tensor tympani muscles are also called accessory mastication muscles [7]. Strong sounds cause stapedial muscle contraction, which improves auditory discrimination [39], while tensor tympani muscle plays a role in the discrimination of low tones. Tensor tympani muscle also contracts in strong sounds, protecting from sound trauma, and vocalization, chewing, swallowing and facial muscle contraction [40]. Malcontraction of the tensor tympani muscle pulls the ossicular chain medially in the middle ear and thus may alter the aural conductive system [9] causing hyperacousia or hypoacousia even with normal audiometric values [41]. It has also been shown that the tensor tympani muscle is dysfunctional in TMD patients, leading to subjective hearing loss [42]. The tensor tympani and stapedial are antagonistic muscles, and with the tympanic membrane are responsible for the appropriate balance and function of the ossicular chain in the middle ear (malleus, incus and stapes). If the tensor tympani or stapedial contract inappropriately, then the perilymphatic and endolymphatic pressures in the inner ear are changed via the oval window, causing vestibular and cochlear impulse imbalance [43]. Changes in endolymph pressure affect the hair cells of the inner ear, which also results in aural symptoms. Electromyographical studies show that the tensor tympani and tensor and tensor veli palatini work simultaneously during swallowing, leading to ventilation of the Eustachian tube [41] raising the intratympanic pressure. The Eustachian tube is actively opened by the tensor palatini and passively opened by the levator palatini muscle. In TMD, the tensor tympani muscle is hypertonic, impeding this normal mechanism. Such a pathological state may lead to hypoacusia, tinnitius, vertigo, otalgia, otic fullness sensation and otitis media [9,44]. Additionally, TMD patients cannot open their mouths wide to yawn, which leads to poor Eustachian tube function as well [36]. One study reported that ear fullness was experienced by 13.5% of TMD and 4.7% of non-TMD patients [RR = 2.87], while in other series involving older populations the relative risk ratio was 14.0 [28]. Objective tinnitus is caused by the palatine and middle ear myoclonus producing rhythmic movement of the tympanic membrane to the tension of the stapedial and tensor tympani muscle. Tinnitus related to TMD is thought to be produced by the lateral pterygoid muscles and the discomalleolar ligament [36].

Furthermore, every movement of the neck or jaw exerts tension on the carotid sheath, increasing the endolymphatic pressure of the hair cells in the cochlea via impedance of the saccus endolymphaticus' pressure-regulating mechanism, causing tinnitus and vertigo [37]. In bruxism and TMD, the tensor veli palatini dysfunctions and may change the position of tympanic membrane and malleus due to its anatomic association with the tensor tympani [45]. Thus, the velopharyngeal, neck and face movements play a major role in otic-TMD symptomatology [7].

The results of a previous study suggested that TMD peripherally sensitizes the V and VII pairs, leading to tonic spasm of these middle ear muscles. This process may cause low tones hearing loss. We found that severe TMD was associated with low tones hearing loss, while moderate TMD correlated with median tones hearing loss. Furthermore, the degree of hearing loss increased with TMD severity. In a study of 44 TMD patients, ear fullness was recorded in 13.6%, vertigo in 13.6% and hearing loss in 6.8% of the subjects. In that same study, audiometry revealed a sensorineural hearing loss in only one subject, at 28-30 dB [46]. Other studies reported no difference in audiographic findings between TMD and non-TMD patients [47], and that hearing loss was not correlated to TMD severity [48]. It has also been previously reported that TMD symptoms are more common in subjects who present with sudden sensorineural hearing loss [49].

Finally, the fourth theory proposes that psychosocial disorders are responsible for the co-existence of aural symptoms in TMD patients [50].

The limitation of our study is the absence of clinical diagnosis of TMD. No clinical examination or other laboratory findings confirmed or rejected each participant's score according to his anamnestic questionnaire. The tool employed only describes signs and symptoms of TMD which may not be seen as a synonym of TMD presence. Thus, many volunteers of this study could not be diagnosed as TMD patients. For the convenience of the results' interpretation we stratified our subjects in four TMD severity groups referring to the number of TMD signs and symptoms. Nevertheless, the results of several studies based on such data verify the utility of evaluation via only an anamnestic questionnaire [12,28].

## Conclusions

The present study found that TMD severity was associated with gender (more common and severe in women), range of mouth opening, the number of aural symptoms, ear itching, temporomandibular joint ankylosis, temporomandibular joint pain, bruxism and qualitative and quantitative hearing loss. The close embryological, anatomical and functional relationships between the temporomandibular joint and the middle ear may explain the link between aural symptoms and TMD severity. A feature of the present study was the use of audiometry to assess hearing loss in TMD. Finally, this is the first study to measure mouth opening in young Greek adults.

## Competing interests

The authors declare that they have no competing interests.

## Authors' contributions

PK conceived of the study and participated in its design and coordination. AM and VG carried out the measurements and drafted the manuscript. KI carried out the statistical analysis of the study. AZ, PK and GP participated in the interpretation of the data and in the design and coordination of the study. All authors read and approved the final version of the manuscript.

## Pre-publication history

The pre-publication history for this paper can be accessed here:

http://www.biomedcentral.com/1472-6815/11/5/prepub
